# Promoter methylation changes in *ALOX12* and *AIRE1*: novel epigenetic markers for atherosclerosis

**DOI:** 10.1186/s13148-020-00846-0

**Published:** 2020-05-12

**Authors:** Jee Yeon Kim, Bong-Geun Choi, Jaroslav Jelinek, Dae Hyun Kim, Seo Hyun Lee, Kwangjo Cho, Seo Hee Rha, Young Ho Lee, Hyo Sun Jin, Dae-Kyoung Choi, Geun-Eun Kim, Sun U. Kwon, Junha Hwang, Jae Kwan Cha, Sukhoon Lee, Jean-Pierre J. Issa, Jei Kim

**Affiliations:** 1 Department of Neurology and Neuroepigenetics Laboratory, School of Medicine and Hospital, Chungnam National University, Daejeon, South Korea; 2 Biomedical Research Center, Chungnam National University Hospital, Daejeon, South Korea; 3 Coriell Institute for Medical Research, Camden, New Jersey USA; 4 Department of Neurology, Dong-A University Hospital, Busan, South Korea; 5 Division of Medical Mathematics Researches, National Institute for Mathematical Sciences, Daejeon, South Korea; 6 Department of Thoracic and Cardiovascular Surgery, Dong-A University Hospital, Busan, South Korea; 7 Department of Pathology, Dong-A University Hospital, Busan, South Korea; 8 Department of Anatomy, School of Medicine, Chungnam National University, Daejeon, South Korea; 9 Department of Vascular Surgery, Asan Medical Center, Seoul, South Korea; 10 Department of Neurology, Asan Medical Center, Seoul, South Korea; 11 Department of Neurology, Chungnam National University Hospital, 282 Moonhwaro, Joongku, Daejeon, 35015 South Korea

**Keywords:** Atherosclerosis, epigenetics, markers, promoter methylation

## Abstract

**Background:**

Atherosclerosis is the main cause of cardiovascular diseases such as ischemic stroke and coronary heart disease. Gene-specific promoter methylation changes have been suggested as one of the causes underlying the development of atherosclerosis. We aimed to identify and validate specific genes that are differentially expressed through promoter methylation in atherosclerotic plaques. We performed the present study in four steps: (1) profiling and identification of gene-specific promoter methylation changes in atherosclerotic tissues; (2) validation of the promoter methylation changes of genes in plaques by comparison with non-plaque intima; (3) evaluation of promoter methylation status of the genes in vascular cellular components composing atherosclerotic plaques; and (4) evaluation of promoter methylation differences in genes among monocytes, T cells, and B cells isolated from the blood of ischemic stroke patients.

**Results:**

Upon profiling, *AIRE1*, *ALOX12*, *FANK1*, *NETO1*, and *SERHL2* were found to have displayed changes in promoter methylation. Of these, *AIRE1* and *ALOX12* displayed higher methylation levels in plaques than in non-plaque intima, but lower than those in the buffy coat of blood. Between inflammatory cells, the three genes were significantly less methylated in monocytes than in T and B cells. In the vascular cells, *AIRE1* methylation was lower in endothelial and smooth muscle cells. *ALOX12* methylation was higher in endothelial, but lower in smooth muscle cells. Immunofluorescence staining showed that co-localization of ALOX12 and AIRE1 was more frequent in CD14(+)-monocytes than in CD4(+)-T cell in plaque than in non-plaque intima.

**Conclusions:**

Promoter methylation changes in *AIRE1* and *ALOX12* occur in atherosclerosis and can be considered as novel epigenetic markers.

## Background

Atherosclerosis is the primary cause of cardiovascular diseases including ischemic stroke and coronary heart disease [1]. Demographic characteristics such as age, sex, and race and established risk factors such as hypertension, diabetes, hyperlipidemia, and smoking are considered predictive markers for atherosclerosis and cardiovascular diseases [2]. Several genetic polymorphisms including alterations in apolipoprotein E [3] and angiotensin-converting enzyme [4] have been suggested as potential genetic markers for atherosclerosis; however, subsequent studies have not supported these findings [5, 6]. In particular, the effect of environmental factors and aging on the development of atherosclerosis has been difficult to investigate owing to the known risk factors and specific genetic polymorphisms.

DNA methylation is one of the epigenetic mechanisms causing the gene silencing without genetic polymorphisms [7]. Gene-specific promoter DNA methylation changes may result from environmental effects [8] and through aging [9]. Atherosclerosis is a typical age-related disease that has been targeted to understand the influence of aging and environmental conditions through epigenetic approaches [10]. After initial reports on promoter hypomethylation in superoxide dismutase [11] and hypermethylation in estrogen receptor α [12] and β [13], several genes with the cellular function of cell proliferations, inflammatory action, and atherosclerosis development were suggested as candidate markers with different promoter methylation signatures [14, 15]. However, the gene-specific promoter methylation causally related with the pathogenesis of atherosclerosis are still insufficient to explain the epigenetic pathomechanisms in the development of the disease [10, 14, 15].

The aim of the present study was to identify and validate specific target genes that undergo promoter methylation in atherosclerotic plaques in order to develop diagnostic markers.

## Results

**Fig. 1:**
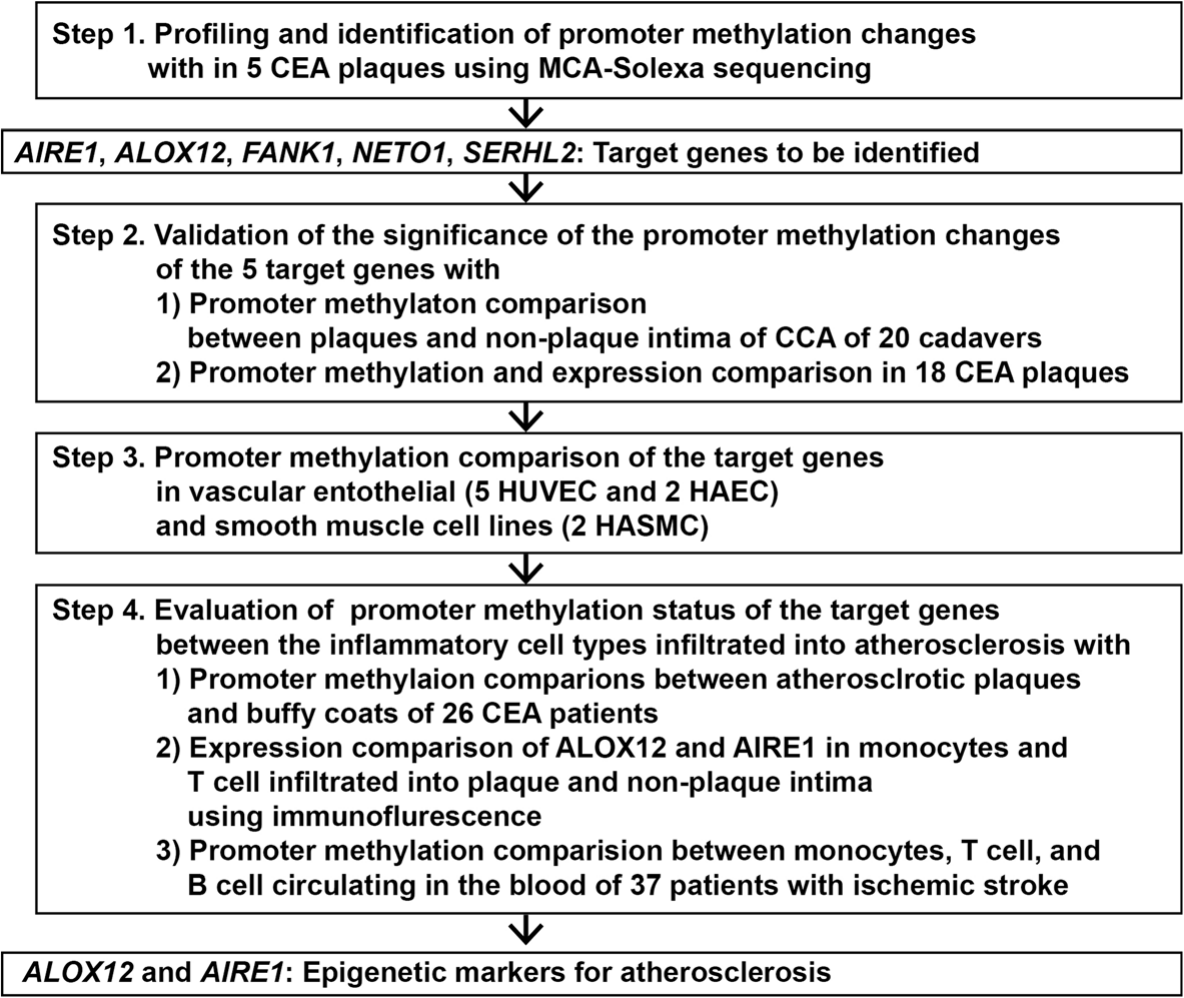
Flow diagram for the present study to profile and identify gene-specific promoter methylation changes related with atherosclerosis. CCA common carotid artery, CEA carotid endarterectomy, MCA methylated-CpG island amplification, HUVEC human umbilical vein endothelial cell, HAEC human aortic endothelial cell, HASMC human aortic smooth muscle cell

The present study was performed in four steps: (1) profiling and identification of promoter methylation changes in atherosclerotic tissues; (2) validation of promoter methylation changes of genes in plaques by comparison with non-plaque intima; (3) evaluation of promoter methylation status of genes in vascular cellular components composing atherosclerotic plaques; and (4) evaluation of promoter methylation differences between inflammatory cell types infiltrated into atherosclerotic plaques (Fig. 1).

### Profiling and identification of gene-specific promoter methylation changes in carotid endarterectomy (CEA) plaques

In the first step of the present study, we initially identified 3275 sequenced tags uniquely mapped to CEA plaques after methylated-CpG island amplification (MCA)-Solexa sequencing. Among the sequenced tags, we selected 61 tags located within 1000  bp upstream from the transcription start site of the specific genes. After excluding 20 repeated sequences and 25 no-CpG islands, we finally identified 16 sequenced tags located in the promoter CpG islands of specific genes (*AIRE1*, *ALOX12*, *APC2*, *FANK1*, *GOLGA7B*, *KIF17*, *MNX1*, *NETO1*, *RIPK4*, *RNASET2*, *RNF126*, *SEC14L2*, *SERHL2*, *SERPINE2*, *VIM*, and *WDFY1*) (Additional file 1: Figure S1A). Upon methylation-specific polymerase chain reaction (MSP) evaluation of the 16 genes in the five CEA plaques used for MCA-Solexa sequencing (Additional file 1: Figure S1B), only five genes (*AIRE1*, 33.8 ± 7.3%; *ALOX12*, 28.2 ± 20.8%; *FANK1*, 53.6 ± 7.4%; *NETO1*, 8.2 ± 18.3%; *SERHL2*, 29.6 ± 5.9%) showed methylation bands in the five CEA plaques used for profiling (Additional file 1: Figure S1C).

### Validation of promoter methylation changes in the five identified genes

**Fig. 2:**
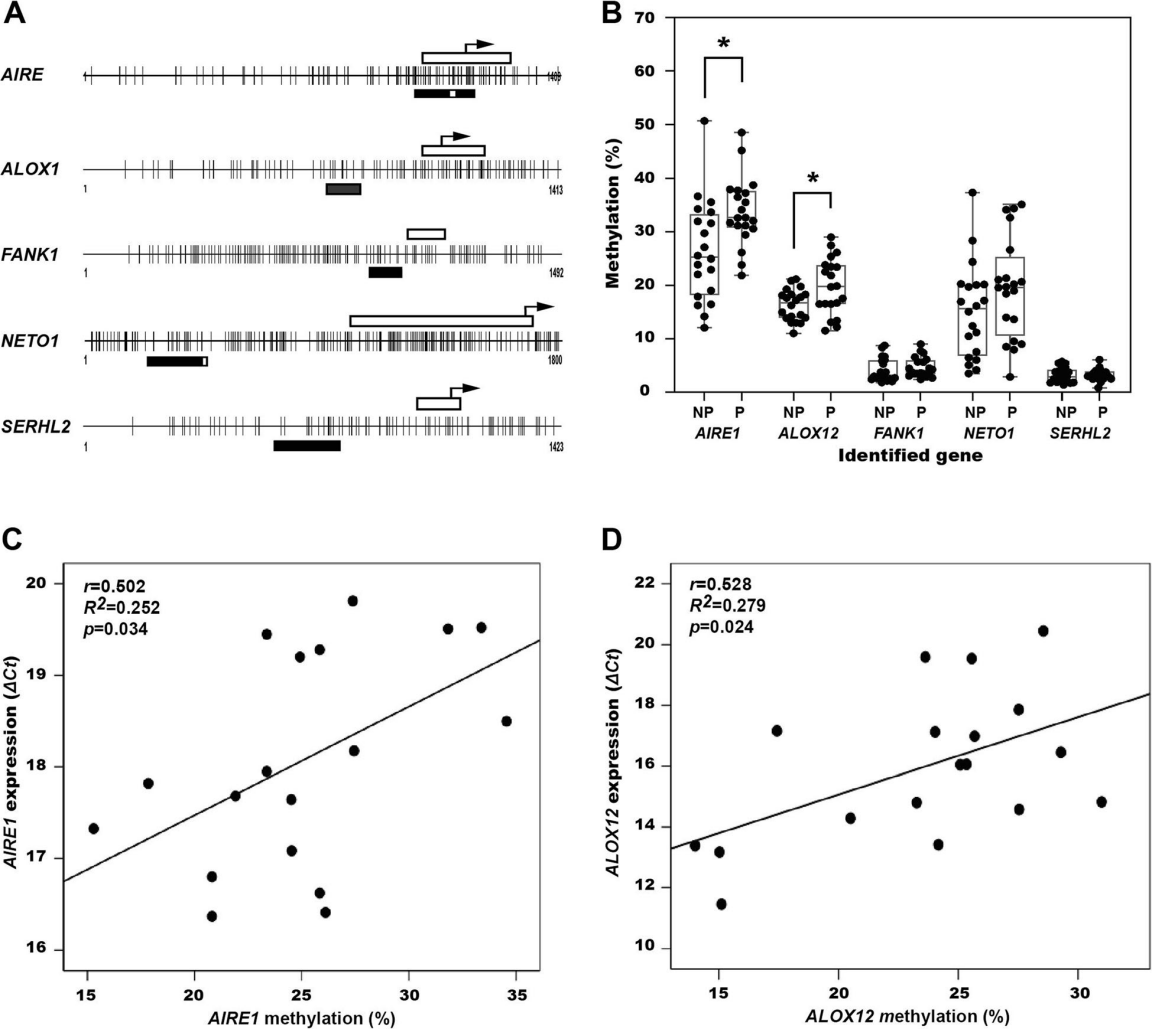
Promoter CpG islands and regions for the pyrosequencing of the five target genes identified after methylation-specific PCR (**a**) and methylation levels of the genes in plaques (P) and non-plaque (NP) intima of common carotid arteries in 20 cadavers (**b**). Correlation between promoter methylation and expression of *AIRE1* (**c**) and *ALOX12* (**d**) in 18 carotid endarterectomy plaques. Open bar, exon 1 region; closed bar, the regions targeted for bisulfite pyrosequencing; open bar in the middle of closed bar; sequencing region, arrow; transcriptional start site of each gene, *p < 0.05 on paired *t* test

In the next step, we tried to validate the promoter methylation changes occurring in atherosclerotic plaques rather than in non-plaque intima. We compared the promoter methylation status of the five genes between the plaques and non-plaque intima harvested from the common carotid artery (CCA) of 20 cadavers using pyrosequencing (Fig. 2a). Promoter methylation was significantly greater in plaques in *AIRE1* (plaques, 33.7 ± 6.4%; non-plaques, 26.3 ± 9.4%, *p* = 0.006) and *ALOX12* (plaques, 19.8 ± 5.3%; non-plaques, 16.3 ± 2.3%, *p* = 0.013) than in non-plaque intima, but in *NETO1* (plaques, 19.4 ± 9.3%; non-plaques, 15.1 ± 8.8%, *p* = 0.429), there was no statistically significant difference (Fig. 2b). Promoter methylation in *FANK1* (plaques, 4.7 ± 1.9%; non-plaques, 4.2 ± 2.2%, *p* = 0.146) and *SERHL2* (plaques, 3.3 ± 1.4%; non-plaques, 3.2 ± 1.1%, *p* = 0.879) was less than 5% and no significant difference between plaques and non-plaque intima was observed (Fig. 2b).

Next, we compared whether the increase in promoter methylation levels of the five genes were related to the decrease in expression of these genes in atherosclerotic tissue. In the correlation analysis performed with 18 CEA plaques, the ΔC_t_ value from real-time RT-PCR increased with an increase in promoter methylation in *AIRE1* (*r* = 0.502, *R*^2^ = 0.252, *p* =  0.034, Fig. 2c) and *ALOX12* (*r* = 0.528 *R*^2^ = 0.279, *p* = 0.024, Fig. 2d); however, no significant correlations were observed between promoter methylation and gene expression for *FANK1* (*r* = 0.004, *R*^2^ = 0.000, *p* = 0.986), *NETO1* (*r* = 0.187, *R*^2^ = 0.35, *p* = 0.430), and *SERHL2* (*r* = 0.185, *R*^2^ = 0.34, *p* = 0.436).

### Evaluation of promoter methylation of the five genes occurring in vascular endothelial and smooth muscle cells

**Fig. 3:**
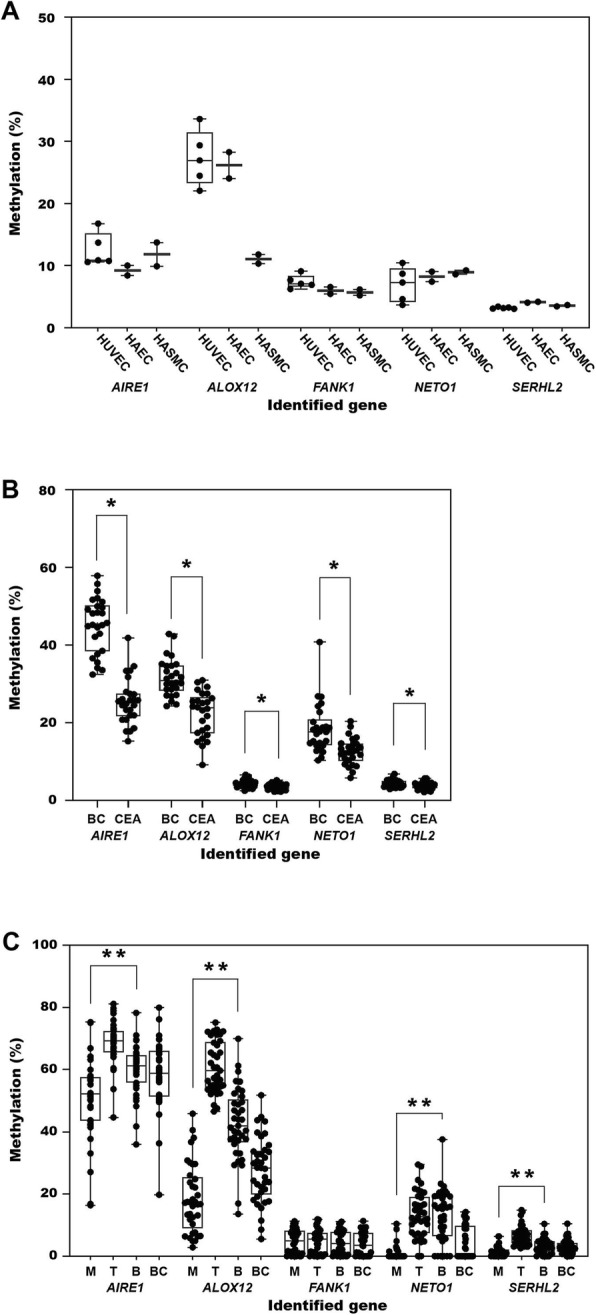
Comparison of promoter methylation status of the five target genes between vascular endothelial and smooth muscle cells (five human umbilical vein endothelial cells [HUVEC], two human aortic endothelial cells [HAEC], and two human aortic smooth muscle cell [HASMC] cell lines, (**a**) between carotid endarterectomy (CEA) plaques and buffy coats (BC) of blood of 27 CEA patients (**b**), and monocytes (M), T cells (T), B cells (B), and buffy coats (BC) of blood of 37 ischemic stroke patients (**c**). *p < 0.01 on paired *t* test, **p < 0.01 on analysis of variance analysis

Atherosclerotic plaques are composed of smooth muscle cells infiltrated with media from the vascular wall as well as inflammatory cells infiltrated from the blood. The infiltrated cells could individually influence the methylation levels measured in the atherosclerotic plaques. In the third step of the present study, we tried to evaluate the promoter methylation characteristics of the five genes in vascular endothelial and smooth muscle cells. In the tested vascular cell lines, the five target genes showed different promoter methylation patterns. *AIRE1* (HUVEC, 12.5 ± 2.7; HAEC, 9.2 ± 1.1; HASMC, 11.8 ± 2.7) and *NETO1* (HUVEC, 6.9 ± 2.8; HAEC, 8.2 ± 1.1; HASMC, 8.9 ± 0.5) showed lower methylation in the vascular endothelial and smooth muscle cell lines. *ALOX12* (HUVEC, 27.3 ± 4.5; HAEC, 26.1 ± 3.0; HASMC 11.1 ± 1.0) also showed lower methylation in smooth muscle cells but higher methylation in endothelial cells. *FANK1* and *SERHL2* had < 10% of methylation in all the tested cells (Fig. 3a).

### Evaluation of promoter methylation of the five genes occurring in inflammatory cell types infiltrating into atherosclerotic plaques

In the fourth and final step, we tried to evaluate whether the promoter methylation patterns of the target genes were different in inflammatory cells infiltrating into atherosclerotic plaques. We first compared the methylation differences between atherosclerotic plaques and buffy coats of blood of 26 CEA patients. Of the five genes, *AIRE1* (buffy coats, 49.3 ± 8.9; CEA plaques, 26.7 ± 6.3, *p* < 0.001), *ALOX12* (buffy coats, 33.3 ± 11.5; CEA plaques, 21.7 ± 5.4, *p* < 0.001), and *NETO1* (buffy coats, 16.8 ± 8.7; CEA plaques, 11.1 ± 3.9, *p* < 0.001) showed significantly lower promoter methylation in CEA plaques than in buffy coats (Fig. 3b).

**Fig. 4:**
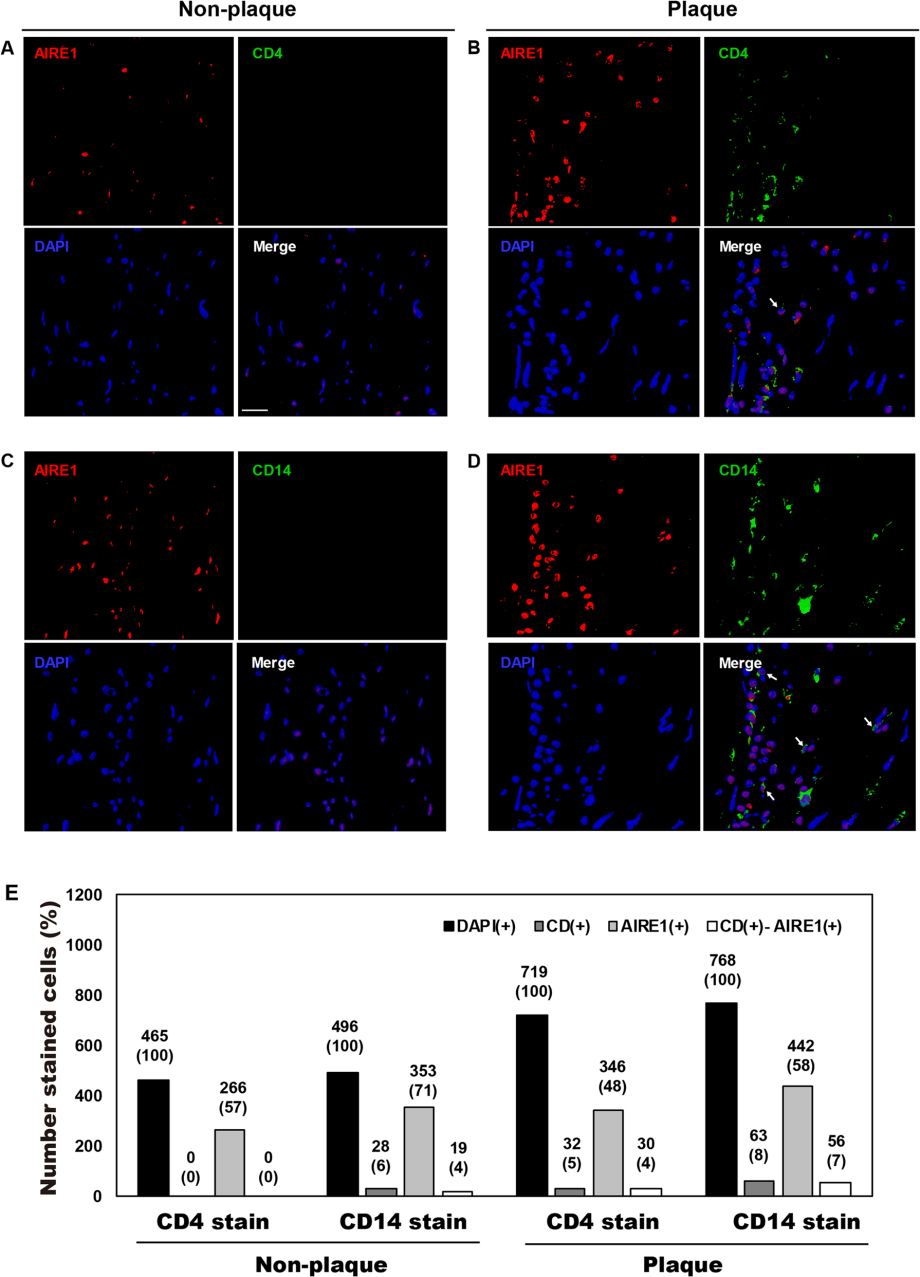
Immunofluorescence staining for AIRE1 and infiltrated CD4(+)-T cells and CD14(+)-monocytes in atherosclerotic plaque and non-plaque intima of common carotid artery of a 54-year-old man. White arrow, cells co-stained with CD14 and AIRE1 antibodies

**Fig. 5:**
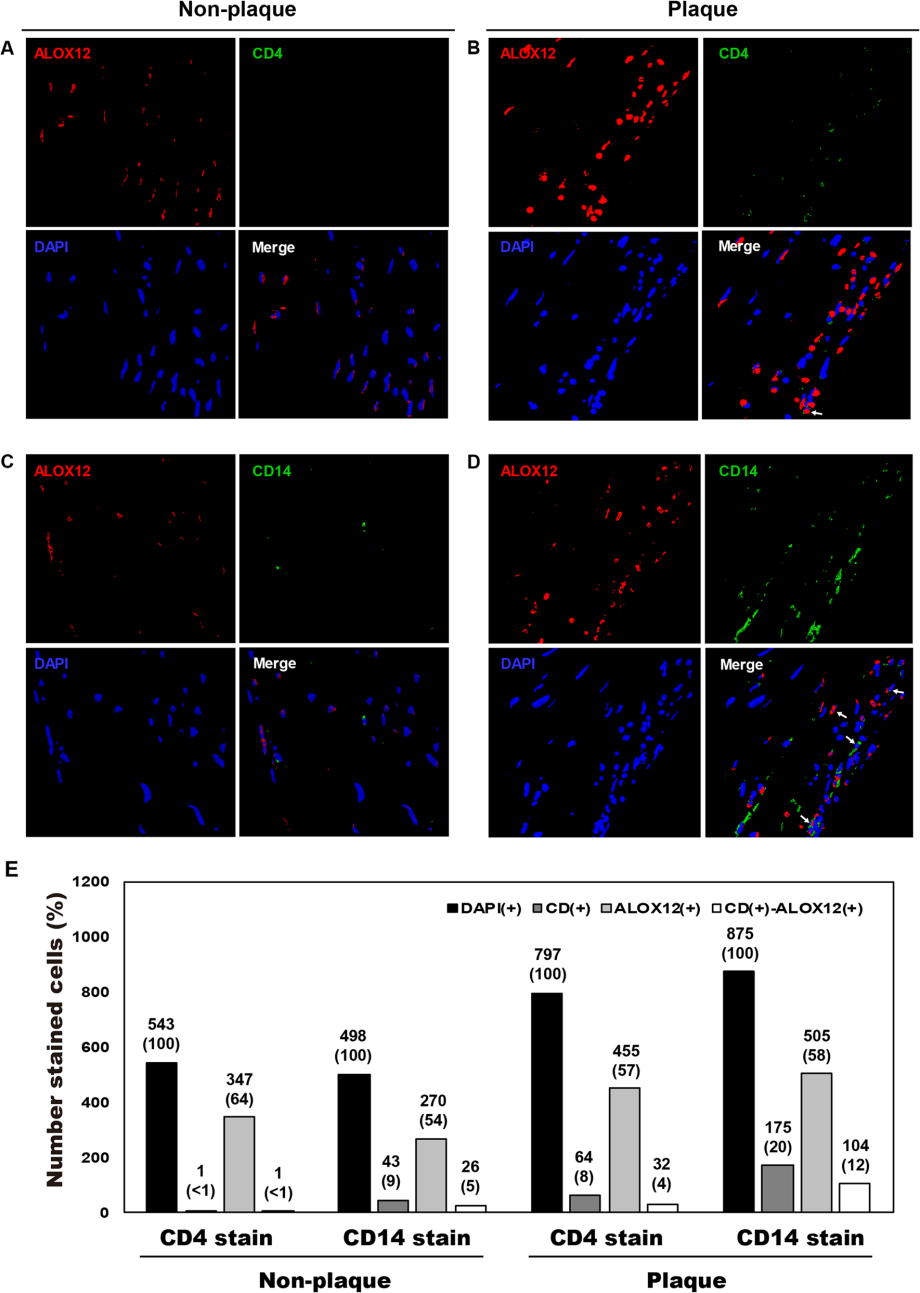
Immunofluorescence staining for ALOX12 and infiltrated CD4(+)-T cells and CD14(+)-monocytes in atherosclerotic plaque and non-plaque intima of common carotid artery of a 54-year-old man. White arrow, cells co-stained with CD14 and ALOX12 antibodies

Next, we tried to evaluate expression differences of the target genes in the inflammatory cell types infiltrated into atherosclerotic plaques using immunofluorescence. AIRE1 and ALOX12, which showed significant differences between atherosclerotic plaques and non-plaque intima  of cadaver, were selected among the five genes. T cells and monocytes were also stained as inflammatory cell types infiltrated into atherosclerotic plaques. In the comparison of expression differences of target genes in inflammatory cell types, expression of AIRE1 (Fig. 4) and ALOX12 (Fig. 5) were different between CD4(+)-T cells and CD14(+)-monocytes in plaque and non-plaque intima of a CCA. CD14(+)-monocytes were more common than CD4(+)-T cells in both non-plaque (CD4[+], < 1%; CD14[+], 1–9%) and plaque regions (CD4[+], 2–8%; CD14[+], 5–20%). In immunofluorescence staining of target genes, ALOX12 was stained in 54–64% and AIRE1 in 48–71% of the total number of DAPI-stained cells in plaque and non-plaque intima. In the evaluation of co-stained cells with antibodies for inflammatory cells and individual target genes, the number of CD14(+) cells co-stained with AIRE1 (plaque, 7% of DAPI[+]-cells; non-plaque intima, 4%; Fig. 4e) and with ALOX12 (plaque = 12%; non-plaque intima = 5%; Fig. 5e) were more frequent in plaque than in non-plaque intima. The number of CD4(+) cells co-stained with AIRE1 (plaque = 4%, non-plaque intima = 0%; Fig. 4e) and ALOX12 (plaque = 4%; non-plaque intima = < 1%; Fig. 5e) were more frequent in plaque than in non-plaque intima, but the co-stained cells were less than 4% of DAPI(+)-cells in both tissues.

Finally, we evaluated promoter methylation differences in the five target genes using inflammatory cells circulating in the blood. We compared the promoter methylation of the five genes among monocytes, T cells, and B cells sorted from buffy coats of 37 ischemic stroke patients (age, 72.8 ± 6.1; men to women = 24:13; transient ischemic attack [TIA] = 6, lacunae or branch atheromatous ischemic stroke = 31, Additional file 2: Table S1). *AIRE1* (monocytes, 47.5 ± 11.0; B cell, 59.8 ± 6.8; T cell, 66.7 ± 5.0, *p* < 0.001) and *ALOX12* (monocytes, 18.5 ± 11.6; B cell, 45.1 ± 12.1; T cell, 59.0 ± 8.1) showed significantly lower methylation levels in monocytes than in B (*p* < 0.001) or T cells (*p* < 0.001) on the post hoc comparison (Fig. 3c). In addition, *ALOX12* and *AIRE1* in B cells were significantly higher than in T cells (*p* < 0.001, Fig. 3c). *SERHL2* (monocytes, 2.2 ± 1.9; T cell, 7.1 ± 3.4; B cell, 4.7 ± 2.0, *p* < 0.001) also showed similar methylation patterns of the lowest methylation in monocytes rather than in B cells (*p* = 0.013) and/or T cells (*p* < 0.001) and significant different methylation between the three cell types (*p* < 0.001) on the post hoc analysis, even though the methylation level was < 10% in all three cell types. *NETO1* (monocytes, 1.4 ± 2.6; T cells, 13.9 ± 7.0; B cells, 16.7 ± 7.7) showed significantly lower methylation in monocytes than in T or B cells (*p* < 0.001) without significant differences between T and B cells (Fig. 3c). *FANK1* methylation (monocytes, 7.7 ± 2.5; T cells, 8.0 ± 2.1; B cells, 7.6 ± 2.2, *p* = 0.846) was < 10% in all three inflammatory cells without a significant difference between them. Buffy coats showed the promoter methylation level taking the mean of the three inflammatory cell types in all five genes (Fig. 3c).

## Discussion

The present study identified promoter methylation changes occurring at *AIRE1* and *ALOX12* as potential epigenetic alterations in the etiology of atherosclerotic plaques. Genome-wide profiling using DNA pretreated with restriction endonucleases, bisulfite modification, or affinity enrichment are suggested to reveal gene-specific changes in promoter methylation related to atherosclerosis [16]. The present study utilized Solexa sequencing with MCA amplicons generated after digestion with *Sma*I/*Xma*I restriction enzyme to profile DNA methylation from CEA plaques. Although MCA theoretically encompasses only 80% of human CpG islands [17], the two genes identified from the present MCA-Solexa sequencing provide unique epigenetic evidences for developing atherosclerotic plaques.

Atherosclerotic plaques and normal intima should be simultaneously obtained from the same individual to validate gene-specific epigenetic alterations profiled in atherosclerosis. However, it is not easy to harvest atherosclerotic plaques and normal intima from the same human body to study the epigenetic characteristics of atherosclerosis. Even though the present study did not use vascular tissues and cells harvested from the same human body as samples, the gene-specific promoter methylation differences between the inflammatory and vascular cells exhibited unique gene-specific epigenetic characteristics that develop in individual cellular components composing the atherosclerotic plaques.

We initially identified *AIRE1*, *ALOX12*, *FANK1*, *NETO1*, and *SERHL2* as possible target genes for epigenetic alteration from the MCA-Solexa sequencing. To verify the significance of the promoter methylation alterations of the five identified genes, we first evaluated the methylation differences of the genes between the plaque and non-plaque regions harvested from the CCA of 20 cadavers. Of the five genes, *AIRE1* and *ALOX12* had significantly higher promoter methylation in plaques than in non-plaque intima of CCA. Although the formalin-fixed cadaver CCA was of limited use in assessing gene expression, the two  gene-specific promoter methylation differences between the plaques and non-plaque intima of CCA support epigenetic contributions to atherosclerotic plaques. The expression changes by the increase of the promoter methylation of *AIRE1* and *ALOX12* were verified using CEA plaques.

Then, we evaluated the promoter methylation differences of the five genes between the cellular components composing atherosclerotic plaques. On comparison between atherosclerotic plaques and buffy coats collected from same CEA patients, *AIRE1*, *ALOX12*, *FANK1*, and *NETO1* of the five genes showed higher promoter methylation in buffy coats than in CEA plaques. Next, we compared the promoter methylation differences of the five genes between inflammatory cell types composing buffy coats of other 37 ischemic stroke patients. *AIRE1* and *ALOX12* showed significantly lower methylation in monocytes than in T and B cells. These findings showed that buffy coats could present the promoter methylation level of *AIRE1* and *ALOX12* taking the mean of the three cell types, but individual inflammatory cells had different epigenetic characteristics. In particular, monocytes had significantly lower promoter methylation of the two genes rather than the levels of T or B cells as well as mean of the buffy coats. On the immunofluorescence stain, CD14(+)-monocytes more frequently infiltrated into both plaque and non-plaque intima rather than CD4(+)-T cell. And CD14(+)-monocytes co-stained with ALOX12 and AIRE1 in plaque lesions were about two times more frequent than in non-plaque intima. The increased number of CD14(+)-ALOX12(+)- and -AIRE1(+)-monocytes relative to the CD4(+)-ALOX12(+)- and -AIRE1(+)-T cells supported the lower promoter methylation of the target genes in monocytes circulating in the blood. Immunofluorescence staining showed that the gene-specific epigenetic status and activation of the five genes differed between the inflammatory cell types circulating in the blood and those that infiltrated atherosclerotic plaques.

In vascular cells, *FANK1*, *NETO1*, and *SERHL2* showed less than 10% and *AIRE1* had about 10% of promoter methylation. Instead, *ALOX12* had over 25% in endothelial cells, but about 10% of methylation in smooth muscle cells. These findings exhibited that vascular cells composing atherosclerotic plaques had lower methylation levels in all five target genes than inflammatory cells. In particular, lower methylation of *ALOX12* in smooth muscle than endothelial cells suggested different epigenetic roles between the vascular cells composing atherosclerotic tissues.

Changes in promoter methylation in *ALOX12* and *AIRE1* in atherosclerotic plaques indicated epigenetic alterations of the inflammatory reactions developing in atherosclerosis. Lipoxygenase (ALOX) pathways catalyze the conversion of arachidonic acid to leukotrienes and lipoxins [18, 19], which are important mediators of inflammation [20, 21]. Pro- and anti-atherogenic activity of ALOX5 and ALOX15 pathways have been reported previously [22, 23]. ALOX12 activation has also been observed in atherosclerosis [24], vascular smooth muscle cells [25], and platelets in the peripheral blood [26]. To explain how the ALOX pathways are related to the development of atherosclerosis, polymorphisms in *ALOX5*, *12*, and *15* have been suggested [27]. The *ALOX12* methylation differences between monocytes and T and B cells suggested that epigenetic alterations in monocytes might be a separate activation mechanism in atherosclerosis development. Further well-designed evaluations are required to clarify the epigenetic mechanisms underlying ALOX pathways in the development of atherosclerosis in the future [28].

The concept of autoimmunity has been proposed as an inflammatory mechanism during the development of atherosclerosis [29]. *AIRE1* was first reported to be responsible for autoimmune polyendocrine syndrome type 1 via regulation of autoimmunity determining thymocyte tolerization [30] and the regulation of B cell and T cell responses to antigenic stimuli [31]. Changes in promoter methylation in *AIRE1* in atherosclerotic plaques might hence provide epigenetic evidence related to the inflammatory reactions developing in atherosclerosis in humans.

*NETO1* is a transmembrane protein regulating synaptic transmissions in the retina and the brain [32] by regulating glutamate receptors such as kainate receptors and NMDA receptors [33]. *FANK1* plays a role in the late meiosis in the testis [34] or anti-apoptosis [35], and *SERHL2* plays a role in normal peroxisomal function in the skeletal muscles [36]. Although the three genes have unique functions in numerous tissues, their roles in atherosclerosis or cardiovascular diseases are unclear. Further studies are required to investigate the changes in promoter methylation in *NETO1*, *FANK1*, and *SERHL2* in the development of atherosclerosis.

The present study established a proof-of-concept for the identification of *AIRE1* and *ALOX12* as potential epigenetic markers for the development of atherosclerosis. Well-controlled future studies are required to overcome the limitations of the present study. These include studies with a larger sample size of atherosclerotic tissues and patients to resolve the limitation regarding generalization of our present results owing to a small sample size. To consider the present epigenetic evidence to identify cardiovascular risk, certain phases have been recommended for prospective validation and evaluation of clinical utility and outcomes as novel markers for cardiovascular diseases [2]. In particular, demographic and established risk factors for the development of atherosclerosis will be simultaneously analyzed with the present epigenetic alterations in a larger number of tissues and patients in future studies. Furthermore, to resolve the limitation in obtaining atherosclerotic tissues and control the normal intima from the human body, surrogate tissues are necessary for future studies. Inflammatory cells circulating in the peripheral blood constitute the primary cellular components infiltrating the intima and contribute to the development of atherosclerosis [37]. So, the circulating inflammatory cells can serve as potential surrogate tissues when studying individual epigenetic characteristics related to atherosclerosis. Future studies are required to determine whether circulating inflammatory cells actually function as surrogate tissues for atherosclerotic tissues and to investigate epigenetic associations between circulating inflammatory cells and atherosclerotic tissues.

## Methods

### Plaque and buffy coat collection from CEA patients

We used two sets of atherosclerotic plaques obtained after CEA. The first set of plaques was obtained from five patients who underwent CEA at Asan Medical Center from March 1 to July 31, 2007, and was used to profile methylation changes. The second set was obtained from 27 patients after CEA at Dong-A Medical Center from December 1, 2015, to September 30, 2017, and was used to validate the significance of the profiled changes. All CEA plaques were immersed overnight in 5 volumes of RNALater (Thermo Fisher Scientific Inc., Carlsbad, CA, USA) immediately after being harvested from each patient and stored in liquid nitrogen until DNA and/or RNA extraction. Buffy coat containing mononuclear cells was separated from the peripheral blood, which was simultaneously obtained from the second set of CEA patients just before the CEA operation. DNA from 50 mg of CEA plaques and 100 μL of buffy coats were extracted with a kit (DNeasy Blood & Tissue Kit, Cat. no. 69506, Qiagen, Valencia, CA, USA) in accordance with the manufacturer’s instructions. All CEA plaque and buffy coat samples were stored in Human Bio-Resource Bank of Asan and Dong-A Medical Centers with informed consent.

### Plaque and non-plaque intima collection from CCA of cadaver

We obtained atherosclerotic plaques and non-plaque intima dissected from individual CCA of 20 cadavers (male to female = 14:6, mean ± SD age = 63.1 ± 18.8 years). Plaques elevated in the intimal layer were distinguished from the surrounding non-plaque intima through naked-eye observation and were clearly dissected along plaque margins. Plaques and non-plaque intima were harvested by peeling them from the media layer of the CCA and stored at − 80 °C until DNA extraction. The plaques and non-plaque intima were also subjected to hematoxylin and eosin staining. DNA from the formalin-fixed tissues was extracted using 100 mg of plaques and non-plaque intima of cadaver CCA with a previously reported protocol [38] and suspended in 100 μL of 1× TE buffer (pH 8.0). Vascular tissues from cadavers were provided by the Human Resources Center of the Department of Anatomy, School of Medicine, Chungnam National University.

### Cultures for vascular endothelial and smooth muscle cell lines

To compare the promoter methylation differences of the identified genes with the CEA plaques, we used five human umbilical vein endothelial cell (HUVEC, Cat. no. C-005-5C, Lot no. 1774129, GIBCO; Cat. no. C-015-5C, Lot no. 1391153, GIBCO; Cat. no. PCS-100-010, Lot no. 70006858, ATCC; Cat. no. PCS-100-010, Lot no. 70008844, ATCC; Cat. no. PCS-100-013, Lot no. 80616172, ATCC), two human aortic endothelial (HAEC, Cat. no. PCS-100-011 lot. no. 64389694 and 70008309, ATCC) and two human aortic smooth muscle (HASMC, Normal cat. no.: PCS-100-012 lot no. 70008916 and 80126171, ATCC) cell lines. HUVECs and HAECs were cultured in M199 growth media (Cat. No. 31100035, Gibco) containing less than 20% fetal bovine serum (Cat. No. 12483-020, Gibco), 2% human serum, 2 mmol/L of l-glutamine (Cat. No. 25030-081, Gibco), and 50 μg/mL of endothelial cell growth supplement (Cat. No. 356006, BD Bioscience). HASMCs were cultured in medium 231 (Cat. No. M-231-500, Gibco) with smooth muscle cell growth supplement (Cat. no. S-008-5, Gibco). All endothelial and smooth muscle cell lines were subcultured for up to five passages, and DNA was extracted using a kit (DNeasy Blood & Tissue Kit, Qiagen) and used for the methylation evaluations.

### Inflammatory cell isolation from the peripheral blood of ischemic stroke patients

We separated monocytes, T cells, and B cells from the peripheral blood of 37 patients, who were admitted as TIA, which was defined as a transient neurological deficit lasting < 24 h and no acute lesion on diffusion weighted magnetic resonance image, and acute lacunar or branch atheromatous ischemic stroke from April 1, 2019 to July 31, 2019 at the Department of Neurology, Chungnam National University Hospital (Additional file 2: Table S1). History of hypertension, diabetes, smoking, and alcohol consumption was checked at admission. Blood tests including hemoglobin A1c (HbA1c), blood glucose, total cholesterol, triglyceride, low-density lipoprotein cholesterol (LDL-C), high-density lipoprotein cholesterol (HDL-C), lipoprotein(a), apolipoprotein-A and apolipoprotein-B, high-sensitivity C-reactive protein (hs-CRP), and homocysteine were checked in the fasting state within 24 h after admission (Additional file 2: Table S1).

To isolate inflammatory cells from the blood, buffy coats were first separated by a density gradient centrifugation using ficoll (Ficoll-Paque PLUS1, Cat. no. 17-1440-02, GE Healthcare). Then, T cells (Dynabeads® CD3, Cat. no. 11151D, Invitrogen), B cells (CD19 pan B, Cat. no. 11143D), and monocytes (CD14, Cat. no. 11149D) were isolated from the buffy coat using magnetic bead kits following the manufacturer’s instructions. DNA from the individual cells were extracted using a kit (DNeasy Blood & Tissue Kit, Qiagen) and stored at − 20 °C until next use.

### Profiling of gene-specific promoter methylation

To profile and identify gene-specific promoter methylations (Fig. 1), we first performed methylated CpG island amplification (MCA) with the DNA extracted from the initial five CEA plaques, as previously reported [39]. The resulting amplicons were used as templates for the subsequent Solexa sequencing analysis, wherein MCA amplicons were digested with *Sma*I. RMCA primer adapters were cleaved with streptavidin-coated magnetic beads. Thereafter, Solexa sequencing adapters were ligated to the amplified *Sma*/*Sma* fragments representing methylated DNA. Single-read Solexa sequencing for 36 bp was carried out using the Illumina/Solexa Genome Analyzer sequencing system as per the manufacturer’s instructions.

### Identification of sequenced tags in promoter CpG islands of specific genes

From the profiled sequenced tags, we first excluded repeated sequences such as long interspersed nuclear elements, short interspersed nuclear elements, or long terminal repeats via the Basic Local Alignment Search Tool (BLAST, http://blast.ncbi.nlm.nih.gov/Blast.cgi?PROGRAM=blastn&PAGE_TYPE=BlastSearch&LINK_LOC=blasthome) and BLAST-like Alignment Tool (BLAT, https://genome.ucsc.edu/cgi-bin/hgBlat). Thereafter, the tags located within the 1000 bp upstream from the transcription start sites of specific genes were selected as candidate genes using BLAT. The sequence within 1000 bp of individual tags located in the promoter regions of specific genes was downloaded from BLAT, and the presence of CpG islands was identified with a definition of > 0.6 of observed/expected ratio and > 50% of GC % using a CpG Island Grapher (http://canna.cau.ac.kr/~yhahn/util/).

### Evaluation of promoter methylation for identified genes

To evaluate promoter methylation status, bisulfite treatment of DNA was first performed using a kit (Zymo Research, Cat. No. D5002, Irvine, CA, USA) with 1 μg of DNA. The bisulfite-treated DNA was stored at − 20°C until further use. The promoter methylation status of 16 genes identified after sequenced tag analysis was evaluated using methylation-specific polymerase chain reaction (MSP) using two primer sets targeting promoter CpG islands in methylated or unmethylated alleles of the 16 genes (Additional file 1: Figure S1A, Additional file 3: Table S2). MSP was performed in 25 μL reaction volumes containing 100 ng of bisulfite-treated genomic DNA, 1× polymerase chain reaction (PCR) buffer, 1.25 mmol/L dNTP mixture, 0.1 mmol/L of forward and reverse primers for methylated or unmethylated alleles, and 1 unit of Taq DNA polymerase. After first denaturing for 5 min at 95 °C and hot-start of Taq DNA polymerase, the reaction conditions were as follows: 35 cycles (95 °C for 30 s, annealing temperature of each gene [Additional file 3: Table S2] for 30 s, 72 °C for 30 s) and final annealing and extension at 72 °C for 5 min. Fifteen microliters of the PCR product was electrophoresed on 6% polyacrylamide gels (Additional file 1: Figure S1B) and visualized via ethidium bromide staining. Methylation levels of these alleles were determined by adjusting the volume ratio of methylated and unmethylated band intensities (Quantity One, ver. 4.0.3, BioRad).

For bisulfite pyrosequencing of the target genes, we used predesigned primer kits for *ALOX12* (PyroMark CpG assay, Cat. No.:PM00065891, Qiagen, Valencia, CA. USA), *FANK1* (Cat. No.: PM00146167), and *SERHL2* (Cat. No.: PM00200746); these comprised forward and reverse primer pairs for PCR and a sequencing primer for bisulfite pyrosequencing (Fig. 2a). We used bisulfite pyrosequencing primer sets for *AIRE1* (AIRE1-F; biotin-5′-GGGTAGTTTTTGTTAGGGTTTTGA-3′ and AIRE1-R; 5′-TCACCCACCAAAACAACTACCTTA-3′ for PCR and AIRE1-S; 5′-TCAAAAAACTCTCACTTCC-3′ for bisulfite pyrosequencing) and *NEOT1* (NEOT1-F; biotin-5′-GTGGGGGGTTTAATTGTAAGAAGT-3′ and NETO1-R; 5′-TCTCTACCTCCCACCCCTTCTTT-3′ for PCR and NETO1-S; 5′-CCCCTTCTTTTCCAT-3′ for bisulfite pyrosequencing) (Fig. 2a). PCR for bisulfite pyrosequencing of each gene was performed in a total volume of 50 μL containing 1× PCR buffer, 0.2 mmol/L dNTP, 1 unit of Taq polymerase, 1 μL (15 ng) of bisulfite-treated DNA, and 0.1 mmol/L of forward and reverse primers for individual genes of which one primer for each gene was biotinylated at the 5′ end. After first denaturing for 5 min at 95 °C with hot-start Taq DNA polymerase, the reaction cycles were as follows: 45 cycles (95 °C for 30 s, 62 °C for 30 s, 72 °C for 30 s), and final annealing and extension at 72 °C for 10 min. Thereafter, bisulfite pyrosequencing was performed, and the methylation levels of the biotinylated PCR products were quantified using a sequencing primer for individual genes and PyroMark Gold Q96 Reagents (Cat. No. 972804, Qiagen, Valencia, CA. USA), using a pyrosequencing machine (PyroMark Q96 ID, Qiagen). Methylation levels in each gene are indicated with mean values from all pyrosequenced CpG sites.

### Evaluation of gene expression

Total RNA from 18 of 27 plaques of the second CEA plaque set was extracted with a RNA extraction kit (NucleoZOL®, Cat. No.740404.200, MACHEREY-NAGEL GmbH & Co, Düren, Germany) and cleaned up with a kit (NucleoSpin®, Cat. No. 74046.50, MACHEREY-NAGEL GmbH & Co) in accordance with the manufacturers’ instructions. Reverse transcription (RT) was performed using a kit (High-Capacity cDNA RT Kit, Cat. No. 4368813, Applied Biosystems, Foster City, CA, USA) with 2 μg of RNA and stored at − 20 °C until use.

The expression of individual genes was evaluated using real-time RT-PCR with RNA extracted from CEA plaques using predesigned primers for the identified genes (TaqMan® Gene Expression Assay, Cat. No.: *AIRE1*, Hs00230829_m1; *ALOX12*, Hs00167524_m1; *FANK1*, Hs00377011_m1; *NETO1,* Hs00371151_m1; *SERHL2*, Hs00367210_m1, ThermoFisher Scientific Inc.) and *β-actin* (Hs99999903_m1) as an internal control in Step-One Real-Time PCR System (Applied Biosystems). Real-time RT-PCR was performed in a total volume of 20 μL containing 10 μL of TaqMan® Gene Expression Master Mix (Cat. No.; 4369016, Applied Biosystems), 1 μL of 20× primer probe mix for individual genes, and 2 μL of cDNA. The cycling conditions were as follows: initial denaturation at 95 °C for 10 min, 40 cycles at 95 °C for 15 s and 60 °C for 60 s. RT-PCR was performed in triplicate for each gene. Expression levels of identified genes were determined with the difference (ΔC_t_) of C_t_ of a specific gene with that of *β-actin*.

### Immunofluorescence staining

To perform immunofluorescence staining, atherosclerotic plaque and non-plaque intima obtained from the aorta of a 54-year-old male cadaver were fixed with 10% formaldehyde, embedded in paraffin, and cut into 4-μm-thick sections. After deparaffinization in xylene and rehydration in an alcohol series, the sections were incubated overnight at 4 °C with primary antibodies including CD4 (Cat. No. ab133616, Abcam, 1:100) for T cell, CD14 (Cat. No. 17000-1-AP, Proteintech, 1:100) for monocytes, and ALOX12 (Cat. No. ab167372, Abcam, 1:100) and AIRE1 (Cat. No. sc-373703, Santa cruz biotechnology, 1:100) for the target genes. The sections incubated with primary antibodies were next incubated with fluorescence-conjugated secondary antibodies at room temperature for 2 h. Then, a final DAPI stain for nuclei followed for 2 min. After mounting, fluorescence images for CD4, CD14, and individual specific proteins (ALOX12 and AIRE1) were acquired using a confocal laser-scanning microscope (TCS SP8, Leica, Wetzlar, Germany), with constant excitation, emission, pinhole, and exposure time parameters.

### Statistical analysis

We used paired *t* tests to compare the differences in percentage methylation of target genes between the plaques and non-plaque intima of 20 cadaver CCAs and between plaques and buffy coats of 27 CEA patients. We used correlation analyses to determine the correlation between promoter methylation and expression of the target genes in 18 CEA plaques. Analysis of variance (ANOVA) tests with post hoc comparison using Bonferroni correction were performed to compare the methylation differences of the target genes between monocytes, T cells, and B cells. All statistical analyses were performed using the SPSS software, version 24.0 (IBM, Chicago, IL, USA).

### Conclusions

The present study identified and validated five genes (*AIRE1*, *ALOX12*, *FANK1*, *NETO1*, and *SERHL2*) with changes in promoter methylation in atherosclerotic plaques. Of these, changes in promoter methylation in *AIRE1* and *ALOX12* suggested epigenetic alterations contributing to atherosclerotic plaques. In particular, epigenetic augmentation of the genes in monocytes might verify the inflammatory mechanism related with atherosclerosis development. These present data reveal novel epigenetic evidence in the development of atherosclerotic plaques and further research is required to fully elucidate the epigenetic contribution to this pathology.

## Supplementary information

**Additional file 1: Figure S1.** Promoter CpG islands of 16 genes profiled after methylated CpG-island amplification-Solexa sequencing with five carotid endarterectomy plaques (**A**), methylation-specific polymerase chain reaction (MSP) results of the five target genes (**B**), and methylation levels of the five genes measured with the five carotid endarterectomy plaques used for the methylated-CpG island amplification-Solexa sequencing (**C**). Open bar, exon 1 region; closed bar, the regions targeted for MSP; arrow, transcriptional start site of each gene

**Additional file 2: Table S1.** Demographics and blood tests of 36 ischemic stroke patients included for the methylation evaluation of the 5 target genes in inflammatory cell of blood.

**Additional file 3: Table S2.** Primer sequences targeting methylated or unmethylated alleles for methylation-specific polymerase chain reactions of the 16 genes identified via methylated CpG-island amplification-Solexa sequencing

## Data Availability

The datasets used and/or analyzed during the current study are available from the corresponding author on reasonable request.

## Abbreviations

CCA: Common carotid artery
CEA: Carotid endarterectomy
HUVEC: Human umbilical vein endothelial cell
HAEC: Human aortic endothelial cell
HASMC: Human aortic smooth muscle cell
MCA: Methylated-CpG island amplification
MSP: Methylation-specific polymerase chain reaction
PCR: Polymerase chain reaction
RT: Reverse transcription
