# Supplementary figures and images for: Promoter methylation changes in ALOX12 and AIRE1: novel epigenetic markers for atherosclerosis
Source: Clin Epigenetics. 2020 May 12;12:66. doi: 10.1186/s13148-020-00846-0 (PMC7218560; doi:10.1186/s13148-020-00846-0)

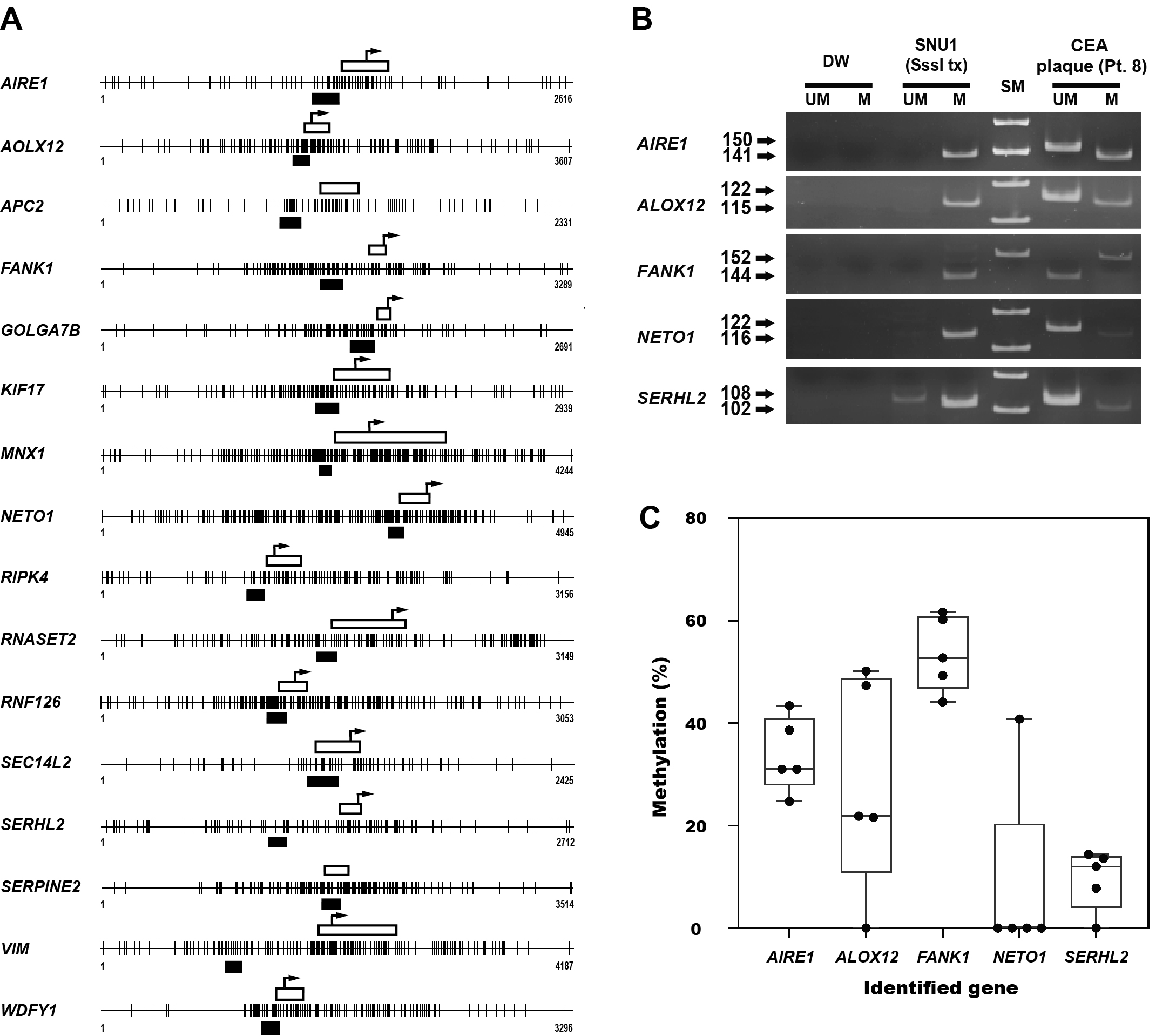

Supplement: Supplementary file 1 — Additional file 1: Figure S1. Promoter CpG islands of 16 genes profiled after methylated CpG-island amplification-Solexa sequencing with five carotid endarterectomy plaques (A), methylation-specific polymerase chain reaction (MSP) results of the five target genes (B), and methylation levels of the five genes measured with the five carotid endarterectomy plaques used for the methylated-CpG island amplification-Solexa sequencing (C). Open bar, exon 1 region; closed bar, the regions targeted for MSP; arrow, transcriptional start site of each gene [file 13148_2020_846_MOESM1_ESM.jpg]
