# Supplementary material for: Promoter methylation changes in ALOX12 and AIRE1: novel epigenetic markers for atherosclerosis
Source: Clin Epigenetics. 2020 May 12;12:66. doi: 10.1186/s13148-020-00846-0 (PMC7218560; doi:10.1186/s13148-020-00846-0)
Supplement: Supplementary file 2 — Additional file 2: Table S1. Demographics and blood tests of 36 ischemic stroke patients included for the methylation evaluation of the 5 target genes in inflammatory cell of blood. [file 13148_2020_846_MOESM2_ESM.docx]

**Supplementary-Table I.** Demographics and blood tests of 36 ischemic stroke patients included for the methylation evaluation of the 5 target genes in inflammatory cell of blood

| Characteristics | Mean (±SD) |
| --- | --- |
| Age | 72.8±6.1 |
| Sex (men:women) | 24:13 |
| Height | 154.3±28.0 |
| Body weight | 60.0±9.5 |
| Body mass index | 23.1±4.9 |
| Risk factors |  |
| Hypertension | 27 (73%) |
| Diabetes | 11 (30%) |
| Smoking | 8 (22%) |
| Alcohol | 9 (24%) |
| Hyperlipidemia | 8 (22%) |
| Ischemic stroke | 4 (11%) |
| Coronary heart disease | 5 (14%) |
| Blood tests |  |
| Hemoglobin | 13.3±1.1 |
| White blood cells | 6387.7±1917.0 |
| Platelet | 214.4±51.0 |
| High-sensitivity C-reactive protein | 3.5±10.2 |
| Homocysteine | 10.2±4.0 |
| Total cholesterol | 155.4±37.7 |
| Triglyceride | 87.4±62.1 |
| High density lipoprotein | 45.7±10.9 |
| Low density lipoprotein | 92.4±29.2 |
| Apolipoprotein A | 119.4±24.9 |
| Apolipoprotein B | 78.2±20.0 |
| Lipoprotein(a) | 21.2±23.3 |
| Hemoglobin A1c | 5.9±0.8 |
| Fasting blood glucose | 135.4±47.6 |
