# Supplementary material for: Promoter methylation changes in ALOX12 and AIRE1: novel epigenetic markers for atherosclerosis
Source: Clin Epigenetics. 2020 May 12;12:66. doi: 10.1186/s13148-020-00846-0 (PMC7218560; doi:10.1186/s13148-020-00846-0)
Supplement: Supplementary file 3 — Additional file 3: Table S2. Primer sequences targeting methylated or unmethylated alleles for methylation-specific polymerase chain reactions of the 16 genes identified via methylated CpG-island amplification-Solexa sequencing [file 13148_2020_846_MOESM3_ESM.docx]

**Table II.** Primer sequences targeting methylated or unmethylated alleles for methylation-specific polymerase chain reactions of the 16 genes identified via methylated CpG-island amplification-Solexa sequencing

| Gene name | Target alleles | Primers | | PCR size (bp) | Annealing temperature (°C) |
| --- | --- | --- | --- | --- | --- |
|  |  | Direction | Sequence |  |  |
| *AIRE* | Methylated | Forward | GTT CGG AGA TTT TTC GAG AGC | 141 | 63 |
|  |  | Reverse | CGC TAT TAT ACC CCC GCG |  |  |
|  | Unmethylated | Forward | TTA GGT TTG GAG ATT TTT TGA GAG T | 150 |  |
|  |  | Reverse | CAC ACC ACT ATT ATA CCC CCA CA |  |  |
| *ALOX12* | Methylated | Forward | TGG GAG CGT TTA AAA TTT GC | 115 | 66 |
|  |  | Reverse | CGA ATC CTA TAC GAT TCC CG |  |  |
|  | Unmethylated | Forward | GAT TTG GGA GTG TTT AAA ATT TGT | 120 |  |
|  |  | Reverse | AAC CAA ATC CTA TAC AAT TCC CA |  |  |
| *APC2* | Methylated | Forward | GGGTTTCGTTTTTCGTATTTAC | 97 | 63 |
|  |  | Reverse | AACAACCAATAACGACCCG |  |  |
|  | Unmethylated | Forward | GGGGTTTTGTTTTTTGTATTTAT | 100 |  |
|  |  | Reverse | ACAACAACCAATAACAACCCA |  |  |
| *FANK1* | Methylated | Forward | CGT TGT TAT TTG TAG CGG | 144 | 63 |
|  |  | Reverse | ACC TAT ACC CCG ACG TCG |  |  |
|  | Unmethylated | Forward | GGT TTT GTT GTT ATT TGT AGT GGT | 152 |  |
|  |  | Reverse | AAA ACC TAT ACC CCA ACA TCA |  |  |
| *GOLGA7B* | Methylated | Forward | TTTAGTATAGCGGATAGCGTC | 130 | 63 |
|  |  | Reverse | ACGACGAACTAAAACCCG |  |  |
|  | Unmethylated | Forward | TTTGTTTTAGTATAGTGGATAGTGTT | 139 |  |
|  |  | Reverse | AATAACAACAAACTAAAACCCA |  |  |
| *KIF17* | Methylated | Forward | TTTAGAAACGGGCGAGTTC | 138 | 64 |
|  |  | Reverse | ACGAAACCTTAAAACAAAAACG |  |  |
|  | Unmethylated | Forward | GGTTTTAGAAATGGGTGAGTTT | 143 |  |
|  |  | Reverse | AAACAAAACCTTAAAACAAAAACA |  |  |
| *MNX1* | Methylated | Forward | CGGGACGGTGATAGGTGC | 96 | 67 |
|  |  | Reverse | AAACTCAACGCGACTTCCG |  |  |
|  | Unmethylated | Forward | GGTTGGGATGGTGATAGGTGT | 105 |  |
|  |  | Reverse | CACTTAAAACTCAACACAACTTCCA |  |  |
| *NETO1* | Methylated | Forward | GGT GTG ATG CGA GCG TTA C | 116 | 67 |
|  |  | Reverse | CAC GCT ACA ACC AAA TCC G |  |  |
|  | Unmethylated | Forward | TTT GGG TGT GAT GTG AGT GTT AT | 122 |  |
|  |  | Reverse | CCC ACA CTA CAA CCA AAT CCA |  |  |
| *RIPK4* | Methylated | Forward | GATTCGTAGCGTTTATTTATTC | 112 | 63 |
|  |  | Reverse | CGTCACTTCCGTAAATCG |  |  |
|  | Unmethylated | Forward | GTAGTGATTTGTAGTGTTTATTTATTT | 123 |  |
|  |  | Reverse | CTATTACATCACTTCCATAAATCA |  |  |
| *RNASET2* | Methylated | Forward | TTTGAGTTAATTACGGGGC | 126 | 62 |
|  |  | Reverse | CCCTACGAACCTACACCG |  |  |
|  | Unmethylated | Forward | TTTTTTGAGTTAATTATGGGGT | 131 |  |
|  |  | Reverse | CACCCTACAAACCTACACCA |  |  |
| *RNF126* | Methylated | Forward | GGTCGTTGTTCGTCGAGTAC | 119 | 66 |
|  |  | Reverse | GTTTACTACTCCCTCGCCG |  |  |
|  | Unmethylated | Forward | TGGGTTGTTGTTTGTTGAGTAT | 123 |  |
|  |  | Reverse | CCATTTACTACTCCCTCACCA |  |  |
| *SEC14L2* | Methylated | Forward | GTTCGTGTTAGAGTTTTTCGC | 151 | 65 |
|  |  | Reverse | AAAACACAAACTCGTCCTCG |  |  |
|  | Unmethylated | Forward | GGGTTTGTGTTAGAGTTTTTTGT | 157 |  |
|  |  | Reverse | TAATAAAACACAAACTCATCCTCA |  |  |
| *SERHL2* | Methylated | Forward | AGA TAG GGT TCG GAA TTG C | 88 | 61 |
|  |  | Reverse | TTC TCA CTC ATC GCT CTC G |  |  |
|  | Unmethylated | Forward | GGA GAT AGG GTT TGG AAT TGT | 98 |  |
|  |  | Reverse | AAC ATT CTC ACT CAT CAC TCT CA |  |  |
| *SERPINE2* | Methylated | Forward | GGGTTGGCGTCGTATTTC | 106 | 65 |
|  |  | Reverse | TCCGCCTACGACTTCTCG |  |  |
|  | Unmethylated | Forward | GTTTGGGTTGGTGTTGTATTTT | 114 |  |
|  |  | Reverse | CCTCTCCACCTACAACTTCTCA |  |  |
| *VIM* | Methylated | Forward | TTTCGTTTCGAGGTTTTC | 123 | 61 |
|  |  | Reverse | AACGATAACCTAAACGACG |  |  |
|  | Unmethylated | Forward | TTATTTTTGTTTTGAGGTTTTT | 132 |  |
|  |  | Reverse | AAAATAACAATAACCTAAACAACA |  |  |
| *WDFY1* | Methylated | Forward | AGGTAATACGGGCGGTGC | 116 | 65 |
|  |  | Reverse | CAACTAACGCCTAAACGAACG |  |  |
|  | Unmethylated | Forward | GTATTTTTAGGTAATATGGGTGGTGT | 128 |  |
|  |  | Reverse | CCATCAACTAACACCTAAACAAACA |  |  |
